# What Can Ribo-Seq, Immunopeptidomics, and Proteomics Tell Us About the Noncanonical Proteome?

**DOI:** 10.1016/j.mcpro.2023.100631

**Published:** 2023-08-11

**Authors:** John R. Prensner, Jennifer G. Abelin, Leron W. Kok, Karl R. Clauser, Jonathan M. Mudge, Jorge Ruiz-Orera, Michal Bassani-Sternberg, Robert L. Moritz, Eric W. Deutsch, Sebastiaan van Heesch

**Affiliations:** 1 Division of Pediatric Hematology/Oncology, Department of Pediatrics, University of Michigan Medical School, Ann Arbor, Michigan, USA; 2 Department of Biological Chemistry, University of Michigan Medical School, Ann Arbor, Michigan, USA; 3 Broad Institute of MIT and Harvard, Cambridge, Massachusetts, USA; 4 Princess Máxima Center for Pediatric Oncology, Utrecht, The Netherlands; 5 European Molecular Biology Laboratory, European Bioinformatics Institute, Wellcome Genome Campus, Cambridge, UK; 6 Cardiovascular and Metabolic Sciences, Max Delbrück Center for Molecular Medicine in the Helmholtz Association (MDC), Berlin, Germany; 7 Ludwig Institute for Cancer Research, Agora Center Bugnon 25A, University of Lausanne, Lausanne, Switzerland; 8 Department of Oncology, Centre Hospitalier Universitaire Vaudois (CHUV), Lausanne, Switzerland; 9 Agora Cancer Research Centre, Lausanne, Switzerland; 10 Institute for Systems Biology (ISB), Seattle, Washington, USA

**Keywords:** Ribo-Seq, mass spectrometry, immunopeptidomics, microprotein, noncanonical ORF

## Abstract

Ribosome profiling (Ribo-Seq) has proven transformative for our understanding of the human genome and proteome by illuminating thousands of noncanonical sites of ribosome translation outside the currently annotated coding sequences (CDSs). A conservative estimate suggests that at least 7000 noncanonical ORFs are translated, which, at first glance, has the potential to expand the number of human protein CDSs by 30%, from ∼19,500 annotated CDSs to over 26,000 annotated CDSs. Yet, additional scrutiny of these ORFs has raised numerous questions about what fraction of them truly produce a protein product and what fraction of those can be understood as proteins according to conventional understanding of the term. Adding further complication is the fact that published estimates of noncanonical ORFs vary widely by around 30-fold, from several thousand to several hundred thousand. The summation of this research has left the genomics and proteomics communities both excited by the prospect of new coding regions in the human genome but searching for guidance on how to proceed. Here, we discuss the current state of noncanonical ORF research, databases, and interpretation, focusing on how to assess whether a given ORF can be said to be “protein coding.”

## Highlights

• Ribo-seq paired with proteomics-based methods optimally detects noncanonical ORFs.
• Data quality and analytical pipelines impact the output of a Ribo-seq experiment.
• Noncanonical ORF catalogs variably report both high- and low-stringency nominations.
• A framework for standardized noncanonical ORF evidence will advance the field.

## Graphical Abstract

**Figure:**
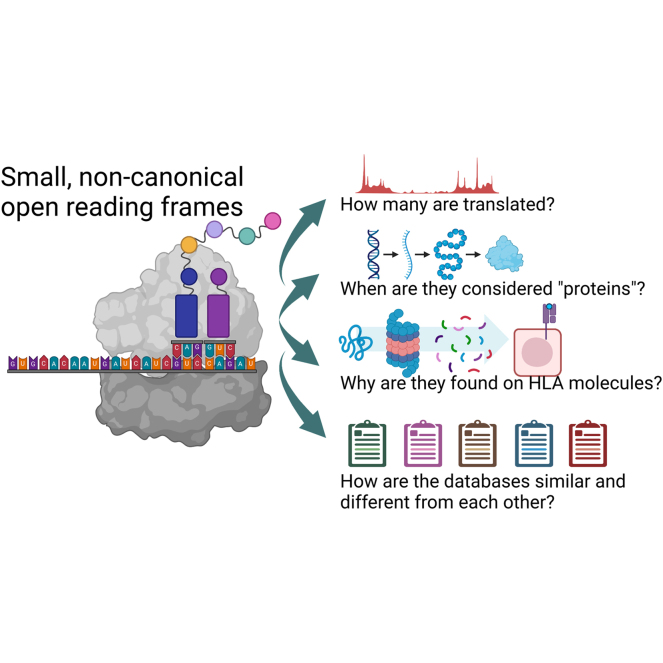

## In Brief

The human genome encodes thousands of noncanonical ORFs along with protein-coding genes. As a nascent field, many questions about them remain: How many exist? Do they encode proteins? What evidence is needed for their verification? Central to these debates has been the advent of ribosome profiling (Ribo-Seq) to discern genome-wide ribosome occupancy and immunopeptidomics to detect peptides presented by major histocompatibility complex molecules. This article synthesizes the current state of noncanonical ORF research and proposes standards for their future investigation and reporting.

Defining the extent of RNA translation in the human genome—and the resulting proteins—has long been a major focus for biomedical research. Approximately 19,500 protein-coding genes, which produce ∼80,000 annotated protein coding isoforms, constitute the canonical proteome (1, 2, 3, 4, 5, 6). Yet, whether this catalog is comprehensive has recently undergone substantial debate spurred by sequencing-based advances in the analysis of ribosome translation, termed ribosome profiling (Ribo-Seq). Based on classical techniques used to isolate ribosome–RNA complexes, Ribo-Seq is an RNA sequencing–based approach that profiles ribosome-protected RNA fragments, precisely defining ORFs actively engaged by translating ribosomes (7, 8). As a tool to detect the translation of RNA, the precision of this methodology is unprecedented: from individual ribosome footprints, the exact codon being translated in a purified ribosome–RNA complex can be determined. Through the sequencing of hundreds of millions of ribosome footprints, a single Ribo-Seq experiment can therefore produce a detailed and accurate representation of a given sample’s translated RNAs, typically identifying ∼11,000 to 12,000 translated genes per sample (9, 10, 11), which is more similar to the ∼12,000 to 13,000 expressed protein-coding mRNAs detected in a given cell type (12) compared with the ∼9000 to 11,000 proteins per sample typically detected in mass spectrometry (MS) methods (13, 14).

**Fig. 1:**
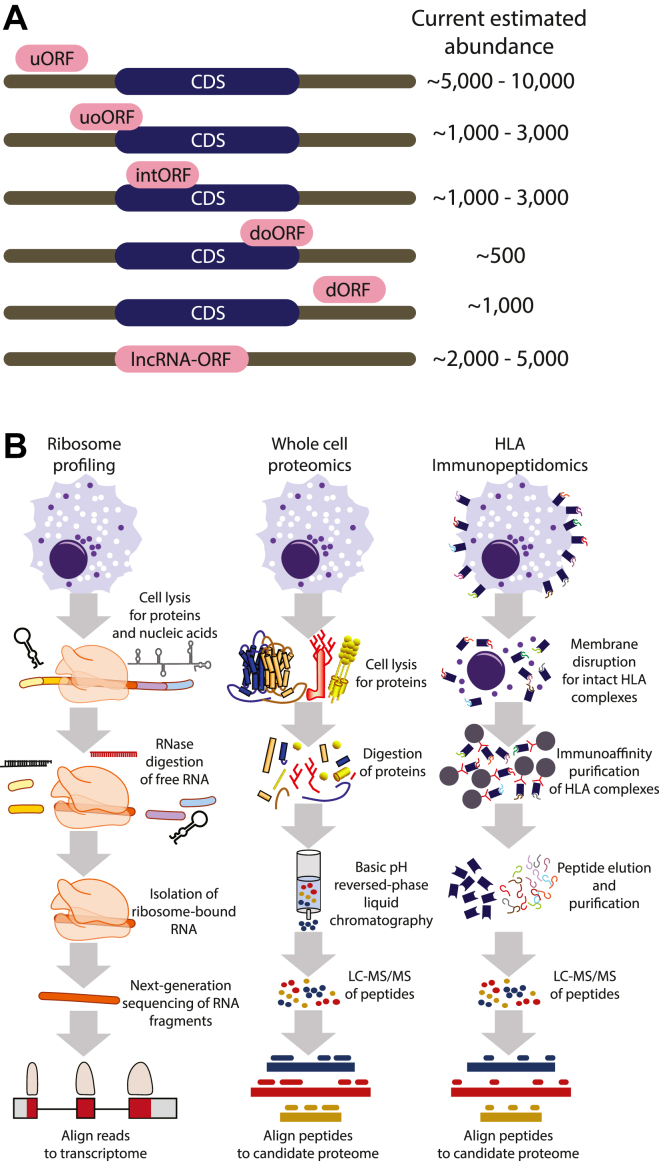
**An overview of noncanonical ORF types and detection methods.***A*, a schematic illustrating the standardized names of noncanonical ORF types, their relationship to known mRNAs, and current estimations of their abundance. *B*, generalized workflows for ribosome profiling (Ribo-Seq), tryptic proteome mass spectrometry, and human leukocyte antigen (HLA) immunopeptidomics. The schematic indicates general properties of sample preparation for these data types. CDS, coding sequence; dORF, downstream ORF; doORF, downstream overlapping ORF; intORF, internal ORF; lncRNA-ORF, ORF residing within an annotated lncRNA; uORF, upstream ORF; uoORF, upstream overlapping ORF.

In addition to confirming known protein coding sequences (CDSs), the high predictive power of Ribo-Seq has unveiled thousands of other genomic sites of ribosome translation. These are most commonly found within known mRNAs (*i.e.*, different reading frames than canonical CDS regions) but also within transcripts annotated as long noncoding RNAs (lncRNAs), pseudogenes, or retroviral elements in the genome (7, 9, 11, 15, 16, 17, 18, 19, 20, 21, 22, 23). Ribo-Seq can also provide clues on previously missed N-terminal in-frame extensions to known CDSs, initiated at sites alternative to the classically annotated initiation codon (24, 25, 26, 27). The nomenclature and estimated abundance of noncanonical ORFs are listed in Figure 1*A*. For clarity, these ORFs are termed “noncanonical” to distinguish them from CDSs included in reference gene annotation—that is, Ensembl-GENCODE—even though their translation, to our knowledge, occurs through mechanisms of ribosome activity similar to that of CDSs. Throughout this text, the term “noncanonical ORF” is therefore defined as any ORF that is not an annotated CDS, an in-frame extension or truncation (either N-terminal or C-terminal), or an in-frame intron retention of an annotated CDS. For our purposes, we will be focusing on upstream ORFs (uORFs), upstream overlapping ORFs (uoORFs), internal ORFs that overlap the CDS but are translated in a different frame (intORFs), downstream overlapping ORFs (doORFs), downstream ORFs (dORFs), and lncRNA-ORFs (as in Fig. 1*A*). We will not discuss in depth ORFs that may be translated from pseudogenes (19), genomic retroviruses (28), or other repetitive sequences (29) (see Limitations section).

Given these observations, the genomics community has been faced with the fundamental question: does the genome actually encode far more than the ∼19,500 protein-coding genes currently accepted as canonical? In response, there have been increasing efforts to corroborate the observations from Ribo-Seq using MS, with the overall conclusion that only a low percentage of noncanonical ORFs are detectable by conventional tryptic proteome methods employing liquid chromatography with tandem MS (LC–MS/MS) techniques (9, 15, 30, 31, 32, 33, 34). Yet, far more noncanonical ORFs appear to be detectable with immunopeptidomic approaches that profile peptides presented by the class I human leukocyte antigen (HLA-I) system (Fig. 1*B*) (34, 35, 36, 37, 38, 39). Moreover, independent of their protein-coding capacity, noncanonical ORFs may serve important roles in the regulation of mRNA translation (40, 41, 42). With these observations at hand, one of the central tasks for the proteomics and genomics communities alike is to develop a consensus understanding on what constitutes sufficient evidence of detection for a noncanonical ORF from each technology and how to standardize these assessments given the limitations of each methodology.

## Types of Evidence for Noncanonical ORFs

Translated noncanonical ORFs can be detected by either Ribo-Seq or LC–MS/MS approaches, with examples of transition to canonical annotated protein-coding genes emerging from both. For example, translation of the signaling proteins, APELA (43), POLGARF (44, 45), TINCR (46), and the cardiac proteins, MYMX (47) and MRLN (48), was first identified using Ribo-Seq, whereas LC–MS/MS data provided the initial evidence for the translation products of uORFs in ASNSD1, MKKS, MIEF1, and SLC35A4 (30, 49).

Together, the combination of Ribo-Seq and LC–MS/MS is a powerful way to identify translated CDSs and ORFs (21, 50, 51, 52). Ribo-Seq does not directly detect proteins but rather provides evidence of ongoing nucleotide translation. By contrast, LC–MS/MS evidence for noncanonical ORFs takes the form of direct detection of peptides. In the case of conventional LC–MS/MS of cellular lysates, these peptides are typically tryptic, meaning they were generated by protein cleavage at the C-terminal side of a lysine or an arginine, or semitryptic, meaning they were generated by protein cleavage at the C-terminal side of a lysine or an arginine at one end of the peptide but not the other. However, many ORFs have now been observed in MS-based HLA-I immunopeptidomics data (18, 34, 36, 38, 53). Here, no tryptic digestion is employed. Instead, peptides containing the HLA-I peptide–binding motifs of the HLA-I allele expressed by a specific cell line or tissue are observed. A variety of lower-throughput approaches have also been used to assess translation of noncanonical ORFs, including generation of custom antibodies, expression of epitope-tagged ORF complementary DNAs, selective reaction monitoring, and radiolabeled *in vitro* translation (9, 17, 54, 55, 56).

While high-quality Ribo-Seq and LC–MS/MS tryptic proteome data on the same sample should be able to identify highly consistent sets of endogenous CDSs, Ribo-Seq is not able to pinpoint the responsible translation event for exogenous proteins, which originate from sources other than the sample’s own genetic material. Similarly, Ribo-Seq cannot detect or predict protein stability, folding, or post-translational modification (PTM). If there is a substantial discrepancy with MS detecting many additional proteins, then the quality of the Ribo-Seq library should be inspected (see later). It should also be noted that Ribo-Seq, like all sequencing-based methods, may not be able to resolve translation events in repetitive genomic regions, such as retrotransposons, pseudogenes, or genes with very high homology.

By contrast, Ribo-Seq will almost always detect many noncanonical ORFs that are not found by proteomics. This is due to several factors: both the nature of the data itself as well as technological differences in the methods that may impact the ability to detect lowly expressed molecules with high confidence. For example, all MS-based proteomics methods lack a PCR amplification step that is present in most nucleotide sequencing–based methods, which enables higher sensitivity at lower sample inputs. Regarding the nature of the data, Ribo-Seq has the ability to identify translating ribosome signatures in an unbiased way, which may confidently find ORFs less than eight amino acids long that are fundamentally challenging to identify by MS (15, 57). In fact, Ribo-Seq can confidently identify an ORF that is simply a start codon followed by a stop codon (*i.e.*, Met∗) because the Ribo-Seq reads remain sufficiently long for unique genomic mapping (58).

Second, since some noncanonical ORFs are located in GC-rich promoters (such as uORFs), these may encode amino acid sequences that are enriched in arginine (CGU/CGC/CGA/CGG codons) and thus would be excessively cleaved by trypsin to small peptides that cannot be uniquely mapped to a single ORF. Whether use of alternative proteases (59) could improve noncanonical ORF detection in whole lysate proteomics is unclear.

## Considerations and Quality Control Steps for the Data-Driven Discovery of Noncanonical Human ORFs

Differences in the nature of Ribo-Seq and LC–MS/MS-based tryptic proteome and immunopeptidome data collection also represent a source of substantial variability in the detection of noncanonical ORFs. Notably, while targeted proteome and immunopeptidome LC–MS/MS approaches may offer improved sensitivity, these require candidate noncanonical proteins of interest to be known prior to analysis. While each method uses high-throughput data generation to profile cellular translation comprehensively, the data have intrinsically different strengths and weaknesses that may result in discordance between them (Table 1).

**Table 1:** Features and characteristics of methods to detect noncanonical ORF translation

Data type	Molecule detected	Digestion step?	Target size of analyte	Number of CDSs detected	Number of ORFs detected	Strengths	Weaknesses
Ribo-Seq	RNA bound within ribosomes	RNase and DNase	28–30 nt	10,000–13,000	2000–200,000	Genomewide	Does not detect proteins directly
						No bias because of trypsin	Cannot detect PTMs
						Detects small and large CDSs	Cannot inform post-translational protein regulation
						Nucleotide-level precision	Analysis pipelines may be discordant
						Defines exact reading frame of ORF	
LC–MS/MS	Tryptic peptides	Trypsin	8–25 amino acids	9000–11,000	10s to 100s	Direct protein detection	High false-positive rate without Ribo-Seq
						Informs protein abundance	Biased against small proteins
						May detect PTMs	Trypsin may bias protein representation
						Proteome-wide	Does not provide nucleotide-level precision
HLA immunopeptidomics	HLA-presented peptide antigens	None	8–12 amino acids	8000–10,000	1000–5000	Direct protein detection	Does not inform protein stability
						Enrichment for low-abundance, strong binders	Does not indicate intracellular abundance
						Proteome-wide	HLA allele expression limits peptide representation
						Can detect unstable translations	Does not provide nucleotide-level precision
						Does not require tryptic sites	

### Ribo-Seq

The quality of a Ribo-Seq dataset is most commonly evaluated using three considerations: codon periodicity, library complexity, and number of canonical CDSs identified.

**Fig. 2:**
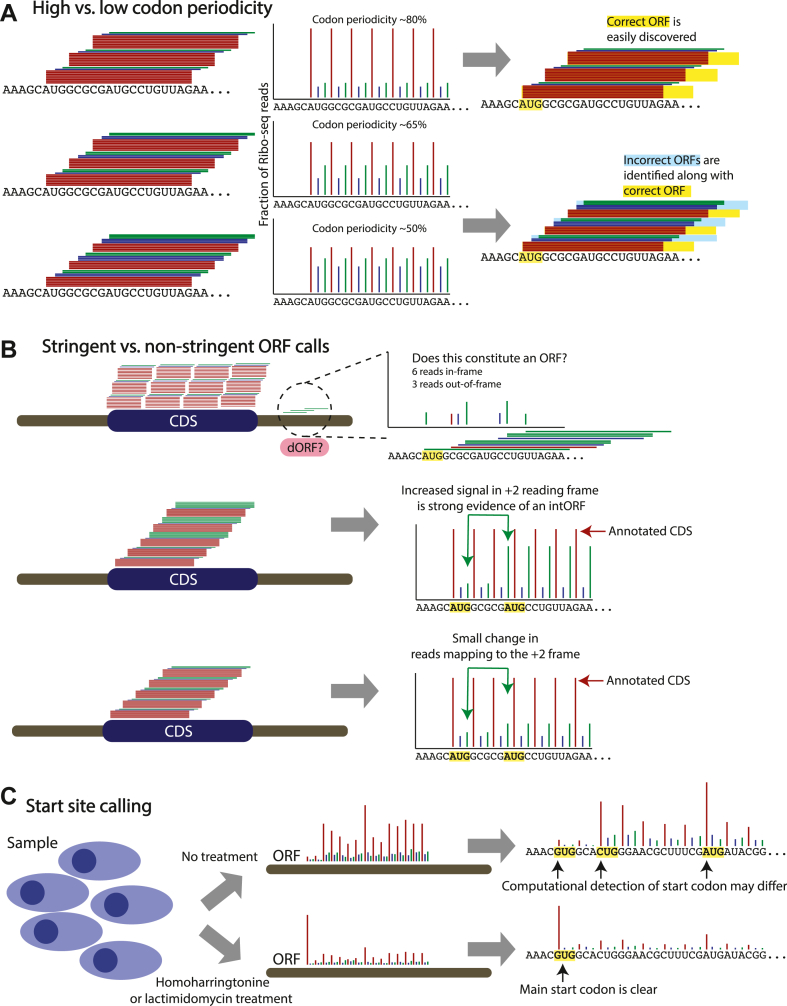
**Quality metrics of Ribo-Seq and stringency of ORF calling.***A*, an illustration showing codon periodicity as a central metric of Ribo-Seq library generation. Three illustrations indicate high-quality, borderline, and poor-quality Ribo-Seq libraries. *B*, an illustration representing high-stringency and low-stringency ORF calling. In the *top* case, a small number of reads map the the 3′UTR of an annotated mRNA, and only two-thirds of those 3′UTR reads support the same reading frame of a potential dORF nomination. In the *middle* and *bottom* cases, a potential intORF has varying read support evidence. The *middle case* shows clear evidence of an intORF by a large increase in reads mapping to the +2 reading frame midway through the CDS. In the *bottom* case, there is a smaller change in the reads mapping to the +2 reading frame. *C*, use of ribosome-stalling drug treatments to clarify translational start sites. Cultured cells are treated with homoharringtonine or lactimidomycin to stall ribosomes at the main translational start site of a given ORF, leading to a clearer resolution of the specific start codon. CDS, coding sequence; dORF, downstream ORF; intORF, internal ORF.

Codon periodicity reflects the percentage of Ribo-Seq reads that correctly identify the known reading frame of CDSs (Fig. 2, *A*–*C*). In a high-quality Ribo-Seq dataset, ≥70% of reads that are between 28 and 30 nucleotides in length map to the correct reading frame of known CDSs. The precise read length that displays the most preferable (the “cleanest”) signal can vary and depends on the sample type and the method of nuclease digestion used to eliminate cellular RNAs not bound within the translating ribosome. Because of limitations of the experimental technique as well as biological variation in ribosome occupancy, a codon periodicity above 90% is typically not attainable (60). A Ribo-Seq dataset with a codon periodicity <60% should ideally not be used for ORF discovery because of challenges with accurate identification of the reading frame (19, 60, 61). A periodicity between 60 and 70% is a gray zone where the data may be used in some cases with increased caution and stringency.

Library complexity refers to the number of unique RNA molecules sequenced and what fraction of these are ribosome footprints that map to CDSs. The challenge with a low complexity library is that the majority of the reads will be PCR duplicates. When the number of initially isolated footprints is limited (*e.g.*, because of low quality of the input material or suboptimal sample processing), ultimately many duplicate copies of this limited number of footprints will be sequenced. This means that deeper sequencing of this library will yield no or only minimally more biologically distinct footprints. Typically, the majority of reads in such low-complexity libraries will come from nonfootprint sources, particularly intergenic and intronic contaminants (*e.g.*, microsatellite repeat elements, ribosomal RNAs, or small RNAs that overlap gene regions), which are unintentionally isolated during the Ribo-Seq procedure because these RNA species are of a similar size to the ribosomal footprint and may have certain RNA structures (62, 63). In general, a Ribo-Seq library with sufficient complexity will have the majority of reads mapping to annotated and novel CDSs. In some cases, such as with degraded samples, there may be substantial intergenic noise or a higher fraction of RNA species that are normally restricted to the cell nucleus but yet still sufficient codon periodicity and library complexity in terms of unique RNA molecules that map to CDSs. Here, the challenge is to achieve sufficient sequencing depth to ensure adequate sampling of unique RNA molecules. While 150 million reads typically suffices for the analysis of a high-quality Ribo-Seq library, a “noisy”—yet usable—library may require very deep coverage (>400 million reads), which is mostly a consideration for the financial cost of the sequencing (60, 64, 65). For human Ribo-Seq libraries, typically 15 to 30% of the sequenced reads can be classified as ribosome footprints, and the rest is often discarded. For a library sequenced to a depth of 150 million reads, that would total to approximately 22.5 to 45 million ribosome footprints—a number comparable to a routinely sequenced RNA-Seq library. Of these, >80% should map to annotated CDSs (60), leaving ∼5 million ribosome footprints for ORF discovery.

The number of known CDSs identified is particularly important when one aims to provide a comprehensive view of all translated ORFs in a sample of interest. This metric relates both to the amount of noise in the library, the periodicity of the footprints, as well as the depth of the sequencing. A sufficiently sequenced Ribo-Seq library for a human sample with high periodicity should detect at least >9000 annotated CDSs and often >10,000 annotated CDSs (9, 10, 11, 18). Human sample Ribo-Seq libraries that do not reach this threshold—despite sufficiently deep sequencing and periodicity—should be used with caution, as the false-negative rate for detecting ORFs will be high (many ORFs will be missed). While Ribo-Seq-based ORF detection tools theoretically have a low false-negative rate, the confidence (false discovery rate [FDR]) with which an ORF or CDS is detected, the number of independent samples in which it can be found, and the translation rate of the ORF should always inform research decision-making. For instance, direct comparison of noncanonical ORF FDRs and translation rates, compared with those of canonical CDSs, can inform both the relative abundance of the ORF’s translation product and the degree of certainty with which the algorithm could nominate it.

Because *de novo* and *ab initio* RNA assemblies are technically challenging with the short nucleotide sequences (28–30 nt) obtained during a Ribo-Seq experiment, analysis of Ribo-Seq data requires alignment of the reads to a reference transcriptome, most commonly Ensembl or RefSeq though custom transcriptomes are also used in some cases. Statistical assessment of a noncanonical ORF nomination is inconsistent across computational methods, with some approaches calculating a *p* value for significance (*e.g.*, RiboTaper (61), ORFquant (10), Ribo-TISH (66), PRICE (67), and RiboCode (68)) and other approaches computing confidence scores (*e.g.*, RibORF (19), Ribotricer (69), ORF-RATER (70)). In addition, these methods are often based on fundamentally different modeling approaches, including hidden Markov (RiboHMM (20)), multitaper (RiboTaper (61)), transformer (DeepRibo, TIS Transformer (71, 72)), support vector machine (RibORF (19)), expectation-maximization (PRICE (19, 67)) models, among others. As such, different methods may be more appropriate for certain research questions, datasets, or desired ORF types.

As a consequence, two different algorithms can have differing ORF outputs for the same gene. This can be due to the level of stringency or the strengths and weaknesses of a particular ORF caller for a certain type of ORF or certain quality of data. For example, some ORF callers cannot detect ORFs with near cognate start codons, whereas others are better suited for the detection of overlapping reading frames where periodic footprint signals are mixed and hard to dissect. Other tools handle alternative splicing better. Depending on the research question, input data quality, species of interest, or annotation goals, combinations of ORF callers followed by curation of called ORFs may be necessary (see later in “How many noncanonical ORFs are there?”).

### HLA-I and HLA-II Immunopeptidomics

In the past decade, interest in HLA-I and HLA-II presented peptides has become widespread across many areas of biomedical research, as a subset of HLA-presented peptides demonstrate antigenic properties and represent a class of potential therapeutic targets (73, 74, 75, 76). The application of HLA immunopeptidomics differs from tryptic proteome protocols, as these methods leverage native lysis buffer and antibody or affinity-tag enrichment steps to isolate HLA–peptide complexes from cell lysates (Fig. 1*B*) (77, 78). The peptides are naturally produced following degradation of endogenously expressed source proteins by cellular proteases and peptidases and the proteasome. As such, no tryptic digestion is used in immunopeptidome analyses, which may enable some noncanonical proteins to be detected by immunopeptidomics even if they cannot generate tryptic peptides. Therefore, regarding detection of noncanonical proteins, HLA immunopeptidome analysis has three advantages over tryptic proteome analysis: (1) each HLA allele has a distinct peptide-binding motif that presents specific subsets of peptides, which can then be detected with MS in the absence of digestion with a protease; (2) the HLA presentation pathway may have privileged access to proteins that are rapidly degraded as the half-life of HLA–peptide complexes (hours) are in general longer than the half-life of rapidly degraded proteins (minutes) (78, 79); and (3) HLA immunopeptidomics broadly samples endogenous proteins from all abundance levels including those from lower-abundance noncanonical ORFs (80, 81, 82). These advantages align with recent studies that have shown higher observation rates of noncanonical proteins in the HLA-I immunopeptidome compared with the tryptic proteome (39, 83).

Similar to tryptic proteome datasets, immunopeptidome datasets require strict quality control steps to ensure the data and analysis are of high quality. Peptide length, the presence of peptide-binding motifs, and predicted binding to HLA molecules coded by specific alleles are common quality control steps in immunopeptidomics workflows. Because HLA-I and HLA-II molecules have unique peptide-binding grooves that accommodate peptides of different lengths, peptide size is an important quality control metric of immunopeptidomics data. Specifically, HLA-I peptides are ∼8 to 12 amino acids long (mostly 9mers), whereas HLA-II peptides are generally 12 to 25mers (77). HLA-II peptides are also typically found in nested sets, while this is not a global feature of HLA-I peptides, and can also be used to quality control HLA-II immunopeptidome datasets. Furthermore, each individual person expresses different HLA alleles with distinct HLA-binding motifs, which influence which peptides are presented. Therefore, it is common to confirm that HLA allele–specific binding motifs of the expressed HLA molecules are present in the immunopeptidome data, and that peptides derived from canonical and noncanonical ORFs in a given dataset are predicted to bind to the expressed HLA molecules to a similar extent. A number of computational approaches (*e.g.*, MHCflurry, NetMHCpan, MixMHCpred, ForestMHC, HLAthena) can be used to both predict HLA peptides and the strength of their binding to various HLA molecules (76, 84, 85, 86, 87, 88, 89). It is important to note that HLA-I binding prediction is currently more accurate compared with HLA-II binding prediction, as HLA-II motifs are more complex and large subsets of diverse HLA-II heterodimers are in the process of being characterized and the associated prediction algorithms are being further improved (90, 91, 92, 93).

Interestingly, peptides derived from noncanonical ORFs are much more abundant in HLA-I datasets compared with HLA-II datasets (18, 34, 36, 38, 39, 53, 94). HLA-I molecules usually present peptides derived from proteasome-mediated degradation of newly synthesized and other cellular proteins, and HLA-I presentation is tightly linked with protein synthesis and degradation rates. In contrast, HLA-II molecules, which are often expressed on professional antigen-presenting cells, present peptides derived from degradation of extracellular proteins that were taken up by the antigen-presenting cells or from endogenous proteins that are destined to be degraded in specialized vacuolar compartments of the endosome–lysosome system. Both HLA-I and HLA-II systems require trafficking to ensure peptide loading in the right compartment. For HLA-I, the peptides themselves are transported into the endoplasmic reticulum by a transporter associated with antigen processing, whereas in case of HLA-II, the source proteins must first reach the acidic compartments for degradation, for example, *via* receptor-mediated internalization or recycling of transmembrane proteins. Hence, the sources of HLA-II–presented peptides are often stable and abundant proteins.

Because of HLA-I binding constraints, and the short length of some noncanonical proteins, a noncanonical ORF is often represented by a single peptide in HLA-I immunopeptidome data, and therefore, additional quality control measures should be taken to support these identifications. To this end, a noncanonical protein subset-specific FDR threshold should be applied to each individual ORF type, rather than a global FDR (83, 95) because noncanonical ORF peptides represent a small fraction (typically <5%) of the overall immunopeptidome and individual ORF types vary considerably in their frequency. Thus, a global FDR can be excessively permissive for a small subpopulation and lead to higher false-positive identifications.

Beyond leveraging known HLA-specific peptide lengths, binding motifs, and subset-specific FDR, there are further quality metrics that can be applied to immunopeptidomics datasets when the focus is the identification of rare noncanonical proteins (96). The gold standard for supporting the identification of noncanonical peptides presented by HLA molecules is by comparing the retention time and MS/MS spectrum of an identified peptide with a synthetic peptide of the same amino acid sequence. However, it is often the case that hundreds of noncanonical peptides are identified in a single HLA-I immunopeptidome experiment, making the synthetic peptide confirmation for all potential noncanonical-derived HLA-I peptides not feasible. To overcome this challenge, it is now possible to compare the observed MS/MS spectra with predicted MS/MS spectra with tools such as Prosit (97). The comparison of the predicted and observed MS/MS spectra provides additional support for noncanonical peptide identification (98, 99). In addition, there are also multiple algorithms that can predict peptide retention times. The predicted retention time, using tools such as DeepLC or DeepRescore, can be compared with measured retention time for all peptides in a sample (canonical and noncanonical), as the correlation between predicted and observed retention time supports the LC–MS/MS identifications of noncanonical-derived peptides in immunopeptidomes (100, 101). Overall, deep learning–based prediction of peptide MS/MS spectra and retention time are powerful tools that help reduce the number of false-positive noncanonical peptide identifications in immunopeptidome datasets.

### Tryptic Proteome LC–MS/MS

Rigorous standards for the analysis of LC–MS/MS tryptic proteome data have been established by the Human Proteome Organization/Human Proteome Project (HUPO/HPP) international consortium, as reviewed elsewhere (102, 103, 104), and these standards remain the expectation for researchers claiming identification of noncanonical ORF peptides (30). For claims of detection of proteins not previously detected, these guidelines require two nonnested and uniquely mapping peptides each of at least nine residues in length with a total extent of at least 18 amino acids and with high-quality peptide-spectrum matches (PSMs) upon manual inspection (30, 102, 104). Peptides may be from different samples but ideally should be reported in the same article to ensure consistency of data analysis, which is consistent with prior HUPO/HPP recommendations (102, 104). These PSMs should be provided in the form of universal spectrum identifiers so that the spectra can be easily examined by others (105).

Yet, consistent application of high-quality tryptic proteome data collection and analysis guidelines remains nonuniform across the research community. Proteogenomic studies looking for noncanonical ORFs without Ribo-Seq data—that is, by predicting and including all ORFs in RNA transcripts—have been plagued by high false-positive rates (30, 49, 106, 107, 108, 109), and initial efforts to inspect early claims of noncanonical ORF peptides concluded that “many of the spectral matches appear suspect” (30).

Moreover, while use of decoys is standard in tryptic proteome experiments to define global FDRs, decoys may be less useful for distinguishing true peptides for noncanonical ORFs. Indeed, Wacholder *et al.* (110) have concluded that decoy bias among noncanonical ORF products leads to inaccurate FDR estimates for short ORFs when decoys are created by reversing the complete protein sequence but not when excluding the initial Met from the reversal. Finally, efforts to identify noncanonical ORFs in tryptic proteome data must account for peptides instead being derived from canonical variants including single amino acid variants and splice-site peptides for alternative isoforms of known CDSs. The use of personalized proteogenomic database searches is not straightforward or used by all in the proteomics community.

Considering these factors, the general experience of the research community is that few noncanonical ORFs are found by conventional tryptic proteome LC–MS/MS analyses, and some of those are ultimately false-positive peptides (111, 112). In some cases, such ORFs are “undiscoverable” by tryptic proteome approaches, either because of the short length of noncanonical ORFs or intrinsic sequence features that do not produce LC–MS/MS observable tryptic peptides. For example, translation of repetitive amino acid sequences (*e.g.*, glycine–leucine) has recently been described (29). Nevertheless, even approaches aimed at enriching for small proteins from cell lysates result in only modest increases in noncanonical ORF detection, rather than exponential increases (33). On the other hand, other enrichment techniques focused on PTMs (*i.e.*, the acetylome, phosphoproteome, and ubiquitylome) have also reported noncanonical proteins and may provide both an alternative method to enrich for noncanonical proteins and also hint toward potential functional relevance of this subset of noncanonical proteins given the cellular roles of those PTMs (83).

Furthermore, data-independent acquisition-MS (DIA-MS) provides a potential opportunity to detect noncanonical ORF-derived peptides that have been reliably detected previously with high-quality spectra obtained with narrow isolation windows from a data-dependent acquisition approach. In DIA-MS, previously identified peptides are more reproducibly sampled by sequentially isolating and fragmenting peptides across the *m/z* range, which decreases stochastic sampling bias toward higher abundant species and may increase the chances of finding rare noncanonical ORFs (113). This approach has been used in conjunction with Ribo-Seq to claim detection of microproteins from noncanonical ORFs (50). Caution should remain with DIA approaches as fragmentation spectra are predominantly a mixture of multiple coisolated peptide ions in broader mass windows, rather than discrete isolated narrow mass ion windows. This results in blended spectra, often containing multiple low-abundance peptide ions, which can confuse DIA algorithms and that make manual verification extremely challenging.

Beyond technical limitations of MS, there are also biological factors that may make noncanonical ORFs less frequently observed in tryptic proteome LC–MS/MS datasets. To this end, there is increasing evidence that points toward intrinsic instability of proteins translated from noncanonical ORFs, resulting in their immediate degradation. Kesner *et al.* (114) used functional genomics approaches to demonstrate that the ribosome-associated BAG6 membrane protein may directly triage hydrophobic noncanonical ORF translations to the proteasome for degradation. Thus, it is possible that many noncanonical ORFs do not generate a stable protein product and might only be observable by immunopeptidomics or in tryptic proteome experiments with inhibition of the protein degradation mechanisms of a cell.

## How Many Noncanonical Human ORFs are There?

The number of noncanonical ORFs encoded in the human genome remains highly speculative. To date, a limited number of human tissues and cell lines have been analyzed by Ribo-Seq, and proteogenomics studies that have aimed to incorporate ORFs derived from these datasets have been difficult to interpret because of numerous false positives. As such, while it is well-established that the human genome contains thousands of translated noncanonical ORFs, whether the precise number is closer to 10,000 or 100,000 remains a matter of debate. A further complication is that different research communities may not use a consistent definition of what types of ORFs we define as “noncanonical.” Yet, while analyses of more cell lines and tissues will certainly uncover additional noncanonical ORFs, there can be variable noncanonical ORF identifications even within analyses of the same cell line. Such variability reflects the equal—perhaps foremost—contribution of different analytical methods for noncanonical ORFs in the estimation of their prevalence.

### The Number of Noncanonical ORFs

Most Ribo-Seq studies focusing on noncanonical ORFs report detection of several thousand ORFs, typically between 2000 and 8000 (9, 11, 15, 16, 18, 19, 20, 21, 51, 61, 115). Interestingly, this range seems relatively stable when comparing studies that employ only a few cell lines and broader analyses looking across many different human tissue types. To consolidate these findings, we have recently participated in an international consortium to aggregate 7264 high-confidence noncanonical ORFs and provided formalized annotations for them within the GENCODE gene annotation database (16). This GENCODE set demonstrates substantial overlap in the identification of certain types of ORFs, such as uORFs, across diverse datasets such as pancreatic progenitors, heart and stem cells, suggesting that perhaps the diversity of several ORF types may not be dramatically larger with the inclusion of more tissue types. In support of this, Ribo-Seq profiling of five human tissue types and six primary human cell types similarly reported 7767 ORFs in total (15). When subsetting this dataset for consistency with the inclusion criteria for the GENCODE catalog (*i.e.*, removing ORFs below 16 amino acids in size, as well as ORFs without an AUG start codon), 2475 of 7767 ORFs remained, of which 1702 (±70%) were represented in the GENCODE catalog as well (supplemental Tables S1–S4).

While these studies have measured and determined noncanonical ORF translation directly from Ribo-Seq data, there are many other databases that have aggregated larger numbers of ORFs from a variety of sources, including both Ribo-Seq and *in silico* predictions. Among these, smProt (n = 327,995 human ORFs (116)), sORFs.org (n = 4,377,422 ORFs across humans, mouse, and fruit flies (117)), RPFdb (118, 119), and smORFunction (n = 617,462 human ORFs (120)) have compiled reported or putative noncanonical ORFs. Notably, OpenProt (121, 122) has two aspects to their database workflow: one that collates all predicted ORFs (n = 488,956) and a second that proposes 33,836 translated ORFs identified by a reanalysis of over a hundred Ribo-Seq datasets with the PRICE pipeline (67). When considering studies that have generated Ribo-Seq datasets to measure noncanonical ORF translation, there are also several efforts that have proffered exceptionally large numbers of directly detected ORFs—specifically, the nuORFdb (34) by Ouspenskaia *et al.* and the Human Brain Translatome Database (123) by Duffy *et al.*, which propose numbers of >230,000 and >75,000 ORFs, respectively.

### Why is There Such Discordance in the Number of Noncanonical ORFs Across Databases?

The interpretation of such dramatically different accounts of noncanonical ORF abundance remains a challenge. Indeed, given that there are currently only ∼60,000 Ensembl genes (including 19,827 protein-coding genes, 18,886 lncRNAs, 4864 small ncRNAs, 15,241 pseudogenes, and 2221 other RNAs in Ensembl, version 109.38), colossal datasets with >200,000 ORFs may be interpreted to suggest that every gene has upward of four distinct ORFs. In practice, these large datasets may include isoform variants (*e.g.*, N-terminal extensions, C-terminal extensions, and intron retentions) that are not part of the reference proteome, and thus the number of noncanonical ORFs may be larger in some databases because of differences in how these isoforms are categorized.

**Fig. 3:**
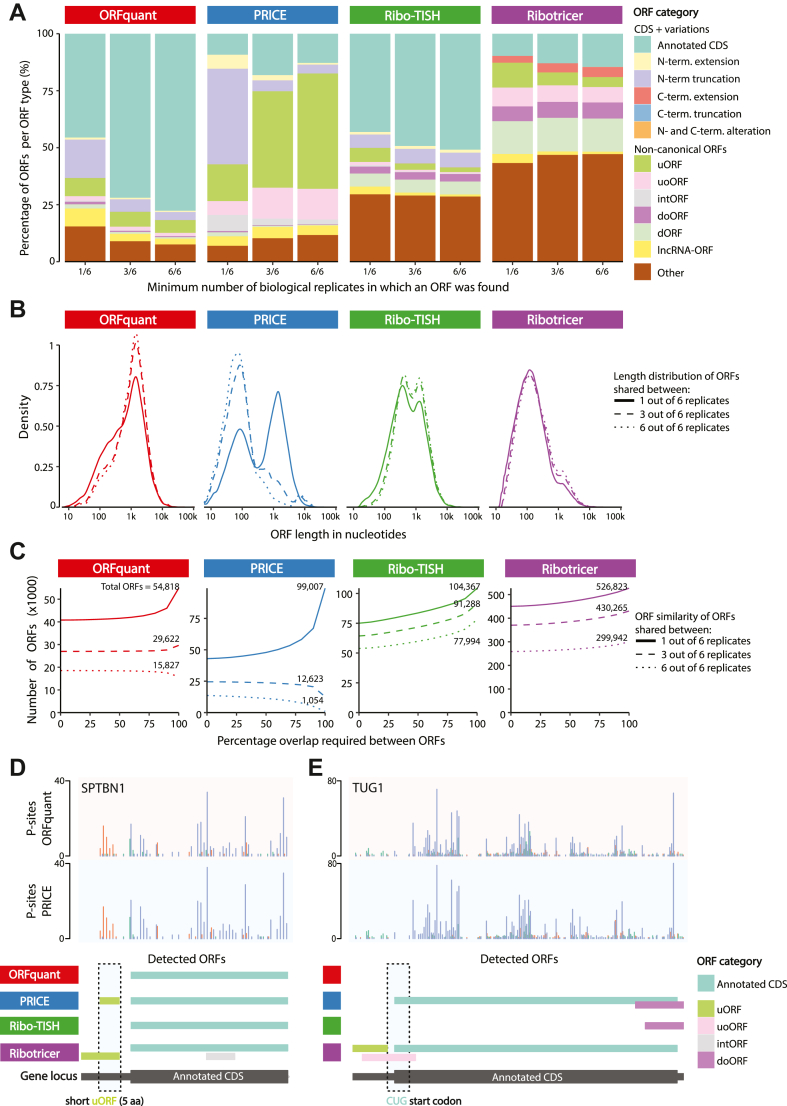
**ORF callers have different specialties and variable performance.***A*, stacked bar plot displaying all detected ORF categories per ORF caller. For each, the percentage of unique ORFs shared between at least one, three, or six replicates is shown. Please note that these are relative contributions to the total number of ORFs. The absolute numbers of ORF identifications can be inferred from *C*. *B*, density plots displaying the distribution of ORF lengths in nucleotides (excluding the stop codon) for unique ORFs shared between at least one, three, or six replicates. *C*, line graphs showing the numbers of unique ORFs detected by each tool shared between at least one, three, or six replicates. The *x*-axis denotes the percentage of overlap used to consider two ORFs being similar or not, with 100% overlap meaning that the detected ORF was fully identical between [x] number of replicates. Please note that the total numbers of ORFs detected per algorithm (*y*-axis) can differ by an order of magnitude. These numbers are given for each line, with numbers reflecting the total ORFs with 100% similarity between replicates (*i.e.*, the end of each curve). *D*, genomic view of a short upstream ORF (uORF) in the *STPBN1* gene indicating that ORF callers have variable affinity for certain types of ORFs. The *top* two tracks show the ribosomal P-site positions derived from the sequenced ribosome footprints, as processed independently from the sequencing data by the deterministic ORF caller ORFquant (*top*; *red shading*) and the probabilistic ORF caller PRICE (*bottom*; *blue shading*). The differently colored P-site bars indicate different reading frames (0, +1, and +2) on the same transcript, with bars in the same color indicating a shared in-frame codon movement by the ribosome. For this visualization, newly found ORF variations of the annotated CDS that could be assigned to predicted noncoding RNA isoforms (*e.g.*, transcript biotype: “processed_transcript”), but matched CDS of SPTBN1 is not displayed. *E*, genomic view of a near-cognate start codon ORF in TUG1. Image and track details as in (*E*) above. CDS, coding sequence.

While sample and data quality likely contribute to the variability in the numbers of noncanonical ORFs in some catalogs, differences in Ribo-Seq data analysis also account for much variation in prospective noncanonical ORFs. For example, biologically, there is some amount of stochastic or pervasive translation across all RNAs, which may relate to leaky ribosomal scanning (124, 125, 126) or transient interactions between ribosomes and RNAs as the ribosomes locate CDSs or RNAs accomplish proper folding (127, 128). Yet, the manner in which computational pipelines process Ribo-Seq data results in ORF calls that may be more or less stringent (Fig. 2*B*), resulting in different proportions of false-positive (stochastic) and false-negative (*e.g.*, sample-specific) ORF calls (60, 129, 130). For example, RibORF (19), which uses a support vector machine and recommends a fixed cutoff score of 0.7, has been shown to produce the highest numbers of ORF calls of any tested algorithm in a recent benchmarking study (131). To confirm these differences directly, we have reanalyzed published high-quality Ribo-Seq data for six biological replicates of pancreatic progenitor cells differentiated from human embryonic stem cells (11) using four common ORF detection pipelines (ORFquant (10), PRICE (67), Ribo-TISH (66), and Ribotricer (69)), observing substantial variability in the number of ORFs called (∼10-fold difference from ∼50,000 to ∼500,000), the types of ORFs called, the length of the called ORFs, and the reproducibility with which ORFs could be detected across all six replicates (Fig. 3 and Experimental procedures section).

**Fig. 4:**
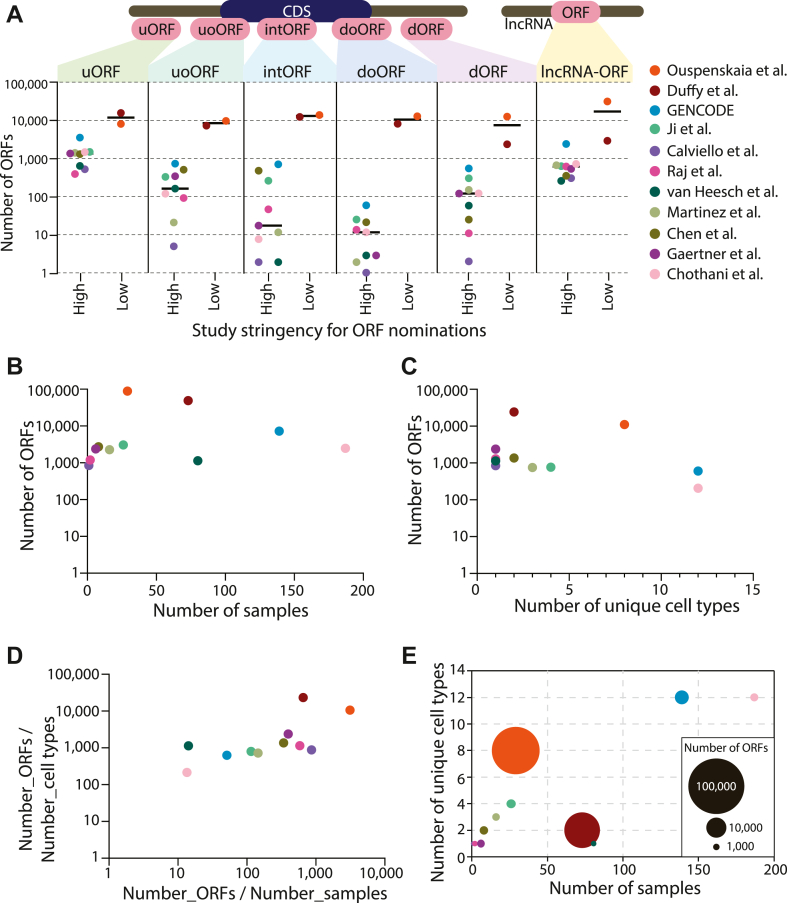
**An analysis of major noncanonical ORF databases.***A*, here, each dot reflects a dataset, and the *Y*-axis uses a log-10 scale to show the number of ORFs included that are ≥16 amino acids long and contain an AUG start codon. The GENCODE catalog reflects the summation of the studies by Ji *et al.* (19), Calviello *et al.* (61), Raj *et al.* (20), van Heesch *et al.* (9), Martinez *et al.* (21), Chen *et al.* (18) and Gaertner *et al.* (11) datasets as described (16). *B*, the number of ORFs per dataset compared with the number of samples profiled by Ribo-Seq. *C*, the number of ORFs per dataset compared with the number of unique cell types profiled by Ribo-Seq. *D*, the ratio of the number of ORFs per cell type compared with the number of ORFs per number of samples for each dataset. *E*, a bubble plot integrating the number of samples, number of different cell or tissue types, and the number of noncanonical ORFs found in each dataset.

There may be specific reasons for the different performance characteristics of each algorithm. For example, the lower stringency of RibORF may be due to the fact that this pipeline considers uniformity of read coverage across the ORF, whereas Ribo-Seq is known to have a 5′ bias to read coverage. Therefore, RibORF may excessively promote intORFs and doORFs since the 5′ ends of these ORFs overlap annotated CDSs, which typically have higher read coverage independent of a periodic footprint signal that matches the correct reading frame. This is evident in nuORFdb (34) and the Human Brain Translatome Database (123): when analyzing the fraction of ORFs with an AUG-start resulting in an ORF ≥16 amino acids, doORFs and intORFs are 173-fold and 18-fold (respectively) higher in abundance compared with other major datasets (Fig. 4, supplemental Tables S5–S8). By contrast, uORFs are only three times more abundant (Fig. 4).

It is also true that different computational pipelines may have different capacity to identify certain classes of noncanonical ORFs. For example, the deterministic multitaper-based statistical inference of significant periodic signal within predicted ORFs as performed by RiboTaper (61) and ORFquant (10) provides high-confidence detection of ORFs with an AUG start codon, but have not, to date, been optimized for non-AUG ORFs. In contrast, the probabilistic algorithm employed by PRICE (67) has enhanced ability to identify very short ORFs and non-AUG ORFs absent from other ORF callers (Fig. 3, *B* and *E*). Yet, when there are neighboring putative initiation codons (*e.g.*, CUG and AUG), PRICE will generate larger numbers of putative ORFs that might require manual curation or further filtering. In addition, since annotated CDSs have generally more abundant Ribo-Seq read coverage, low-abundance out-of-frame reads may be more readily interpreted as an intORF with a non-AUG start codon by PRICE, whereas other ORF callers are less likely to consider these reads as sufficient evidence for a translated ORF. Thus, when applied to biological replicates of the same sample, PRICE produces the least consistent ORF calls compared with other pipelines, independent of initiation codon variability (Fig. 3, *A*–*C*) (131). nuORFdb (34) and OpenProt (122) both employ PRICE in their analysis pipelines. It is important to note, however, that the specific research question being pursued should inform the types of ORF callers used: indeed, deterministic algorithms such as RiboTaper or ORFquant may miss intORFs or overlapping ORFs identified by PRICE because of the difficulty in resolving mixed periodicity signals of overlapping reading frames (Fig. 3*A*).

In summary, depending on the type of ORF one aims to find and the desired inclusiveness of ORFs one aims to output, one ORF caller might be better suited than another. Certain ORF callers outperform others in detecting specific ORF categories such as intORFs (Fig. 3*A*), very small ORFs (Fig. 3, *B* and *D*), or near cognate start codons (Fig. 3*E*), whereas others handle exon–exon junctions and longer ORFs better and/or provide better replicate behavior. These differences then lend to substantially different results when producing noncanonical ORF catalogs (Fig. 4).

### Detection of Translational Start Sites

Determining the translational start site of an ORF remains a nuanced problem. While conventionally proteins have been annotated with AUG start sites, exceptions to this rule have long been known (132, 133), and noncanonical ORFs are more likely to employ non-AUG start sites (125, 134). In a typical Ribo-Seq experiment, identification of translational start sites from Ribo-Seq data is inferred based on two factors: sequencing coverage and the intrinsic restrictions of the computational pipeline (*e.g.*, some algorithms only consider AUG start codons, as discussed previously). Yet, independent of the computational pipeline, there may be gaps in the sequencing coverage that lead to misidentification of the main translational initiation site (Fig. 2*C*). For experiments with cultured cells, use of small molecules that block ribosome elongation, such as homoharringtonine (135) or lactimidomycin (136), enables ribosome accumulation on translational initiation sites, which enables more precise determination of the start codon. Because of the difficulty in identifying noncanonical ORF start sites and the variability in computational approaches to start codon recognition (*e.g.*, Fig. 3*E*), use of homoharringtonine or lactimidomycin with cultured cells is highly recommended. In frozen tissue samples, these compounds are no longer effective.

## How to Select an ORF Sequence Database for MS Data Analysis?

Given the wide differences between the different databases for Ribo-Seq ORFs, one central question is how to use these databases, or which to use for any specific analysis? Because the size of the ORF output in a given database can vary enormously, users should base their decision on what scientific question they intend to pursue and evaluate carefully the suitability of the input Ribo-Seq data quality as well as the stringency with which ORF calling was performed. In general, high stringency databases provide high-confidence Ribo-Seq ORF detections, and thus peptides found mapping to these ORFs are more likely to reflect a true positive result. While these databases reduce false positives, it is at the expense of comprehensiveness, as the existing high stringency databases will yield more false negatives in the MS analysis. Low stringency databases provide a much larger set of Ribo-Seq ORFs but will yield more false positives—because of the lack of support from another orthogonal technique. If the ORFs are accompanied by Ribo-Seq quality metrics, it may be tractable to estimate the proportion of false positives and refilter the ORFs to suit one’s own purposes. These databases will provide a larger candidate search space for peptide alignment and may enable detection of true positive ORFs not present in the high stringency databases. Yet as described earlier, because of the concern for false-positive nominations, ORFs detected by MS searches should be closely inspected to verify integrity of both ORF call and peptide identification, as there will likely be cases of false-positive ORFs being supported by false-positive peptides. Ultimately, certain scientific questions may lend themselves to certain databases: for example, analyses of alternative N-terminal CDS extensions often emphasize non-AUG start sites (24), which may benefit from a Ribo-Seq analysis that employs the PRICE algorithm. Research efforts aimed to identify a maximal space of potential translation events may also favor a lower stringency database, with the caveat that any individual result should receive additional scrutiny. Alternatively, if the goal is to characterize a high-confidence unannotated microprotein, a high stringency database may be more desirable. Likewise, for reference annotation purposes and functional studies, we prefer more stringent workflows that yield reproducible ORF calls across samples (no false positives).

## Are Noncanonical ORFs Proteins?

The term “protein” is conventionally used to refer to an amino acid sequence that produces a molecular structure that plays an intrinsic cellular role in maintaining normal cell biology. While some proteins may be unstable and rapidly degraded under certain conditions (*e.g.*, beta-catenin), most proteins participate in cell biology when present in a stable form. Also, almost all annotated proteins show evidence of evolutionary conservation, structural folding, and domain architecture, and frequently also protein–protein interactions and/or interactions with nucleic acids.

According to this understanding of the term “protein,” it could be inferred that the vast majority of noncanonical ORFs do not encode proteins on the basis that they lack these characteristics. To our knowledge, microproteins from noncanonical ORFs also do not have paralogs within the proteome that might enable inferred protein functions. However, we see two additional considerations. First, it may be incorrect to assume that a protein that exists in the cell—even one that is detectable by MS—is therefore a functional molecule. It could be that the proteome contains a certain amount of nonfunctional translational “noise.” While it is difficult to prove the extent to which such translation occurs in normal cells, evidence from cancer cells shows abundant dysregulation of translation, exemplified by “aberrant” noncanonical proteins that lack evidence for function under normal physiological conditions (34, 35) as well as out-of-frame peptide byproducts of oncogene activity (137).

Second, the classical definition of protein “function” invokes the protein’s role in cellular processes that have been derived over time through evolution, which has been summarized as the maxim that “conservation = function.” This maxim has been central—but not universally required—for gene annotation projects, and the only canonical proteins currently within GENCODE that can be inferred to have evolved *de novo* in human or higher primates were initially detected in cancer cells (*e.g.*, MYEOV (138) and HMHB1 (139)). Even so, evidence for the existence and function of *de novo* proteins under normal physiological conditions is accumulating (57, 140, 141, 142). Nonetheless, it remains true that most noncanonical ORFs display much higher rates of intrinsic disorder, fewer structural features, and lack amino acid constraint across evolution (17, 18, 140, 141, 143, 144, 145, 146, 147, 148, 149). While these features may be observed in diverse annotated proteins (*e.g.*, intrinsically disordered regions of a given protein), their presence is predominant in noncanonical ORFs.

The absence of protein function as a criteria should not determine whether noncanonical ORFs are categorized as translational “noise.” Indeed, the function of many human proteins remains obscure, motivating multi-institutional efforts such as the Understudied Proteins Initiative (150) and the HPP Grand Challenge to define “a function or functions for every human protein” (151). In the case of noncanonical ORFs, because many may only exist as unstable peptides that are presented on the immunopeptidome, the question of whether potential recognition by T cells constitutes a molecular “function” becomes a central and partly philosophical debate for the research community. There is no current precedent to regard major histocompatibility complex presentation as a central “function” of a protein—as opposed to an ancillary observation for a protein that has additional roles in cell biology—and therefore, in the absence of additional experimental data on this question, we are disinclined to consider major histocompatibility complex presentation as proof that a noncanonical ORF has an intrinsic cellular role at this time.

### The Interpretation of Peptide-Level Evidence of Ribo-Seq ORFs

How, then, should one interpret the peptide-level evidence for some noncanonical ORFs? High-quality tryptic proteome LC–MS/MS PSMs that survive rigorous manual inspection are strong evidence of true translation of a noncanonical ORF. With adequate evidence, therefore, tryptic proteome PSMs supporting noncanonical ORFs do indicate the possible existence of a translated protein, and these cases may reasonably be considered to be part of the cell proteome, similar to any other proteins.

When considering the larger number of noncanonical ORFs with peptide-level evidence in HLA immunopeptidomics but not tryptic proteome LC–MS/MS (18, 34, 36, 38, 152), firm conclusions are more difficult to draw. These noncanonical ORFs cannot be said to generate a true protein based on immunopeptidomics alone, considering that the HLA system is expected to present peptides resulting from translation products that are unstable and rapidly degraded, alongside those derived from canonical proteins. Yet, detection of an HLA-presented peptide does verify RNA translation in these cases, which distinguishes them from the majority of Ribo-Seq-detected noncanonical ORFs that are detected in neither tryptic proteome LC–MS/MS nor immunopeptidomics experiments. Therefore, these noncanonical ORFs can at least be said to be confirmed as both translated and presented by the HLA, as opposed to an artifact of the Ribo-Seq protocol.

A related question is how to interpret PSMs matching noncanonical ORFs that are not detected by Ribo-Seq, when the same sample is interrogated using both technologies. Because the sensitivity of Ribo-Seq is generally higher than MS-based methods, and because Ribo-Seq provides nucleotide-level precision for genome mapping, there are three possibilities here: first, these peptides may be false-positive identifications, second, the Ribo-Seq data exhibit a false-negative identification, or third, they may be derived from another source not included in the search space (*e.g.*, aberrant splicing). None of these hypotheses has been rigorously evaluated at this time. One challenge is that many proteomics and immunopeptidomics experiments do not currently generate matched Ribo-Seq data for their samples, and thus it cannot be directly known if Ribo-Seq supports translation of that ORF. When considering unmatched analyses, it is also noted that, at present, proteomics and immunopeptidomics datasets cover a broader range of tissue and cell types than Ribo-Seq datasets.

## A Proposed Framework to Classify the Translation of Noncanonical ORFs

Given the expanding volume of research on noncanonical ORFs, a shared vocabulary for the interpretation of their detection is a critical need in the genomics, translatomics, proteomics, and immunopeptidomics communities. Notably, there has been no formalized initiative to annotate noncanonical ORFs as protein-coding genes by major genome databases, although recent collaborative work has raised this point as a topic of interest (16). Historically, protein-coding genes have been annotated one by one in a manual process of careful data inspection, which may or may not have included protein-level evidence. At this time, noncanonical ORFs detected by tryptic proteome data would potentially be eligible for manual annotation as protein-coding genes. Yet, given the paucity of noncanonical ORFs in tryptic proteome data and their much greater abundance in HLA immunopeptidomic datasets, there is uncertainty about whether most noncanonical ORFs produce proteins in the classical sense, and whether immunopeptidomic evidence is equivalent to tryptic proteome data for the purposes of protein annotation.

We advocate both a cautious but open-minded approach to noncanonical ORF classification, summarized in Table 2. Notably, although most annotated proteins show evidence of amino acid constraint across species and most noncanonical ORFs do not, it is also unquestionably true that at least some proteins are lineage- or species-specific. Thus, we propose that *de novo* translations should be considered for annotation as protein coding. While recognizing that evolutionary analysis is a core part of gene annotation workflows in projects like GENCODE, we have not included conservation or constraint metrics as part of this proposed framework. The framework itself is oriented toward harmonizing subsequent dataset generation and analysis. In practice, it might be applied to classifying published datasets, and it is intended as a helpful tool for candidate prioritization rather than a guarantee that certain ORFs will be annotated by a genome database. We stress that researchers looking to move forward with potential annotation of a protein encoded by a noncanonical ORF should be able to provide the raw LC–MS/MS spectra for review.

**Table 2:** A proposed framework to standardize levels of evidence of noncanonical ORFs

Tier	Required supporting evidence	Standardized outcome
Tier 1A	Tryptic proteome LC–MS/MS (≥2 peptides according to HUPO/HPP criteria)	“Protein candidate.” Consider discussing research findings with genome annotation databases for possible annotation.
	Ribo-Seq^a^	
Tier 1B	HLA immunopeptidomics MS (≥2 observations; multiple high-confidence peptides from multiple distinct sources)	“Presented”
	Ribo-Seq^a^	
Tier 2A	Tryptic proteome LC–MS/MS (≥2 peptides not satisfying HUPO/HPP spacing criteria)	“Detected”
	Tryptic proteome LC–MS/MS (1 peptide)	
	Ribo-Seq^a^	
Tier 2B	HLA immunopeptidomics MS (1 observation)	“Detected”
	Ribo-Seq^a^	
Tier 3	Any HLA immunopeptidomics or tryptic proteome LC–MS/MS evidence without Ribo-Seq^a^ evidence	“Putative,” consider alternative sources
Tier 4	Ribo-Seq^a^ evidence without any proteomic evidence	“Ribo-Seq ORF”
Tier 5	*In silico* prediction of an ORF on an expressed transcript without any Ribo-Seq^a^ or proteomic evidence	“Predicted”

a From credible Ribo-Seq data with quality metrics meeting the guidelines suggested in this article. Ribo-Seq need not be performed on aliquots of the same samples analyzed by proteomics.

Our framework centers proposes these definitions for specific terminology:

• “*Protein candidate*”: a tier 1A noncanonical ORF can be regarded as translated into a protein candidate if it satisfies current HUPO/HPP guidelines for the detection of ≥2 uniquely mapping tryptic proteome peptides, as well as having evidence of translation by Ribo-Seq. Such candidates would be prioritized for further manual review by annotation groups.
• “*Presented*”: A presented noncanonical ORF (tier 1B) is one with multiple lines of evidence for its translation and presentation on HLA molecules. These ORFs are detected with multiple high-confidence peptides from multiple distinct samples for HLA immunopeptidomics data as well as having evidence of translation by Ribo-Seq.
• “*Detected*”: A detected noncanonical ORF is one with evidence of translation by Ribo-Seq as well as evidence of protein production by either (tier 2A) tryptic proteome LC–MS/MS (1 peptide or >1 peptide not satisfying HUPO/HPP guidelines for their spacing) or evidence of protein production by HLA immunopeptidomics with a single PSM (tier 2B).
• “*Putative*”: A putative noncanonical ORF (tier 3) is one with evidence of translation with tryptic proteome LC–MS/MS or HLA immunopeptidomics data but no evidence of translation in Ribo-Seq data. This discrepancy may alert to the possibility of false-positive MS identifications or false-negative absence in Ribo-Seq and therefore requires more investigation.
• “*Ribo-Seq ORF*”: A noncanonical ORF that is only detected in Ribo-Seq data but not elsewhere is considered a “Ribo-Seq ORF” (tier 4). These are likely to be the majority of cases. The number of these ORF nominations may be variable based on the stringency of the Ribo-Seq analysis and/or the quality of the input data.
• “*Predicted*”: A predicted noncanonical ORF (tier 5) is one that is computationally predicted *in silico* on an expressed RNA transcript but without current evidence in Ribo-Seq or MS datasets.

## Experimental Procedures

### Benchmarking and Comparing ORF Caller Performance on Replicate Ribo-Seq Datasets

#### Ribo-Seq Data Processing and Mapping

Ribosome profiling data of late pancreatic progenitor cells obtained from six independent differentiations of H1 human embryonic stem cells (11) were collected from the Gene Expression Omnibus database (GSE144682). For all analyses, the Ensembl primary DNA assembly (GRCh38) and the Ensembl human reference transcriptome (Ensembl v102) were used as reference. Quality control and trimming of the Ribo-Seq reads was done using Trim Galore 0.6.6 with the options “--length 25” and “--trim-n” (153). Next, contaminant RNA and DNA were removed using Bowtie2 2.4.2 by aligning reads to a contaminant file using the default options of Bowtie2 (154). The contaminant-depleted reads were aligned using STAR with the options “--twopassMode Basic,” “--outFilterMismatchNmax 2,” “--outFilterMultimapNmax 20,” “--limitOutSJcollapsed 10,000,000,” “--alignSJoverhangMin 1000,” and “--outSAMattributes All” (155). For PRICE, the option “--alignEndsType EndToEnd” was set as well. Also, the individual bamfiles were filtered using SAMtools 1.12 to exclude reads with a mapping quality lower than 5 (156).

#### ORF Calling With ORFquant

The function RiboseQC_analysis from RiboseQC 1.1 was run in R 4.1.2 with the options “read_subset” and “fast_mode” set to false (157). The output was used by the function run_ORFquant from ORFquant 1.02 in R with the default options (10). ORF calling with PRICE: Before using PRICE, a reference genome was created with the IndexGenome function of the Gedi framework 1.0.2. After the creation of the reference genome, PRICE 1.0.3b was run (67). A filtered list of ORFs detected by PRICE and a list of P-sites (called activity values by PRICE) were extracted from the outputted “orfs.cit” files using the Gedi Nashorn and ViewCIT functions, respectively. Because the start codon prediction is a separate step in the PRICE program, ORF coordinates from both before and after start codon prediction were available. We used the coordinates after start codon prediction. PRICE can also be run in a multisample mode by providing a text file with the bam file locations as input. This mode favors ORFs that occur in all samples during the ORF calling process and would likely enhance the reproducibility of ORF calls between replicates. To keep all ORF callers comparable, we did not use this mode. ORF calling with Ribo-TISH: From Ribo-TISH 0.2.7, the predict function was used to infer ORFs with the option “--longest” set (66). The output file contained only the genomic start and end coordinates and the transcript id of each ORF. The reference GTF was used to determine the exons within each ORF. ORF calling with Ribotricer: The Ribotricer 1.3.3 function prepare_orfs was first used with the options “--longest” and “--min_orf_length 9” (69). The option “--start_codons” was set to include all near cognate start codons with one base difference compared with ATG. Afterward, the function detect_orfs was used with the option “--phase_score_cutoff 0.440.”

#### Comparing ORF Callers

ORF calls were compared between algorithms for the types of ORF categories that were found, in how many replicates they were independently discovered, how ORF differed in length, and how reproducible and similar their detection was based on, for example, the percentage of ORF sequence overlap between replicate ORF calls. Before the analyses, data were converted to GRangesList objects in R with stop codons included in the coordinates. ORF categories were determined by comparing the start and end coordinates, and the transcript id of each ORF with the CDSs in the “gtf.rannot” object created by the ORFquant function “prepare_annotation_files.” ORFs were compared by their overlap, with different thresholds set for the required percentage of overlap. Two ORFs were considered to be similar if the exons of one ORF were fully contained within the exons of a second ORF, both codons had the same stop codon, and the first ORF covered at least the required percentage of overlap of the length of the second ORF. These overlap relations were recursive, such that a parent ORF could be the child of another ORF, and all three would be counted as one unique ORF.

### Comparison of Published Ribo-Seq Datasets

We used publicly available datasets from GENCODE (16), Chothani *et al.* (15), Ouspenskaia *et al.* (34), and Duffy *et al.* (123) for comparisons of published reports of noncanonical ORFs that might encode microproteins. The GENCODE dataset itself is a metaanalysis of data from Ji *et al.* (19), Calviello *et al.* (61), Raj *et al.* (20), van Heesch *et al.* (9), Martinez *et al.* (21), Chen *et al.* (18), and Gaertner *et al.* (11); datasets employed are listed in supplemental Table S1. Source data for these datasets are listed in supplemental Table S2. To facilitate comparisons between studies, we extracted only noncanonical ORFs with a length of ≥16 amino acids and had an AUG start codon. For ORFs using a non-AUG start site, the first internal AUG start codon was identified and the amino acid sequence starting with that internal AUG was included for analysis if the resulting ORF was ≥16 amino acids long. ORFs were then analyzed for their replication across primary datasets. Since the GENCODE list represents a meta-analysis of other individual datasets, the presence of an ORF in the GENCODE list was not used as part of the analysis for ORF replication across primary datasets. Next, ORF calls were associated with one of the following six categories: lncRNA-ORF, uORF, uoORF, internal ORF, doORF, or dORF, according to the schema by Mudge *et al.* (16). Duffy *et al.* used the nomenclature “external” for doORF, and these ORFs were reclassified as doORF for this analysis; they used “internal” for intORFs, which were reclassified as intORFs for this analysis. For lncRNAs, Duffy *et al.* used the term “noncoding,” which included the biotypes “noncoding,” “lncRNA,” “antisense_RNA,” “misc_RNA,” “TEC,” and “processed_transcript,” which were included as part of the lncRNA-ORF designation for this study. For Ouspenskaia *et al.*, we analyzed ORFs according to the authors’ designation of ORF “plotType,” reflecting their final classification. Ouspenskaia *et al.* used the term “3′ dORF” for dORF, “3′ overlap dORF” for doORF, “5′ overlap uORF” of uoORF, “5′ uORF” for uORF, “lncRNA” for lncRNA-ORF, and “out-of-frame” for intORF. Chothani *et al.* reported final ORF types of “dORF,” “doORF,” “ncORF,” “overlap_uORF,” “intORF,” and “uORF.” For Chothani *et al.*, Duffy *et al.*, and Ouspenskaia *et al.*, ORFs that had a final classification of pseudogene were excluded from this analysis; however, these datasets variably reclassified some ORFs on pseudogene transcript biotypes as noncoding or lncRNA, and we did not refilter these ORFs beyond the original reclassifications provided by the authors. ORFs that switch a classification corresponding to a small RNA, tRNA, or rRNA species, such as “rRNA,” “snoRNA,” “tRNA,” “snRNA,” or “miRNA,” were excluded from this analysis. The number of cell types and/or tissue types for analyses of each ORF dataset was extracted from the source publication.

## Limitations

With this work, we have endeavored to clarify how Ribo-Seq can be used for noncanonical ORF research. Yet, our focus has several important limitations. First, the vast majority of—but not all—translated peptides can be traced back to an RNA sequence. There may be peptides that derive from amino acid splicing within the proteasome during protein degradation (158), which would not be detectable in Ribo-Seq data. Second, there are also well-established protein CDSs that are difficult to resolve with Ribo-Seq and do not have optimized computational methods for their quantification. For example, translated pseudogenes, retroviruses, retrotransposons, and paralogous protein-coding genes may have high sequence homology that precludes unique mapping of the short ∼30 bp reads from a Ribo-Seq experiment, although multimapping reads will provide evidence of translation. These cases are not discussed here. This issue of short Ribo-Seq sequencing reads also highlights the potential role for emerging long-read sequencing technologies to enhance detection of noncanonical ORFs on alternative transcript forms (159), which we do not discuss. Finally, each individual’s genome (and particularly each cancer’s genome) has a unique range of germline or somatic single nucleotide variants that will impact the proteome: in this article, we have not addressed the importance of generating personalized reference genomes and proteomes for the analysis of microproteins and noncanonical ORFs.

## Conclusions

The widespread description of noncanonical ORFs has sparked a paradigm shift in the perception of both the human genome and the proteome. Yet, as a field still in its infancy, this area of investigation is plagued by a lack of standardization, which may lead to imprecise analyses, ultimately leading to self-injurious confusion. While the proportion of noncanonical ORFs that encode a functional protein remains to be seen, a large fraction of them can be verified as translated by both MS-based and Ribo-Seq-based approaches. A central effort for the research community is now to build reputable databases and analysis pipelines to ensure rigor in this quickly expanding—and highly exciting—field while also enabling functional studies to proceed with confidence. Here, we have considered the technologies used to detect noncanonical ORFs and attempted to provide a framework for categorizing differing levels of evidence for them. Our work aims to coalesce the research community around a common terminology and shared set of database resources for noncanonical ORFs. Ultimately, we believe that the study of noncanonical ORFs, if pursued with proper precision, will prove invaluable to the global community of biomedical researchers.

## Inclusion and Diversity

We support inclusive, diverse, and equitable research.

## Code Availability

All codes used for these analyses as well as data visualization are available at https://bitbucket.org/vanHeeschLab/orfcaller_comparison.

## Supplemental data

This article contains supplemental data.

## Conflict of interest

The authors declare no competing interests.
