# Supplementary material for: What Can Ribo-Seq, Immunopeptidomics, and Proteomics Tell Us About the Noncanonical Proteome?
Source: Mol Cell Proteomics. 2023 Aug 11;22(9):100631. doi: 10.1016/j.mcpro.2023.100631 (PMC10506109; doi:10.1016/j.mcpro.2023.100631)
Supplement: Supplemental Data [file mmc2.docx]

**Supplementary Materials**

**What can Ribo-seq, immunopeptidomics, and proteomics tell us about the non-canonical proteome?**

**Author list**

John R. Prensner,^1,2^ Jennifer G. Abelin,^3^ Leron W. Kok,^4^ Karl R. Clauser,^3^ Jonathan M. Mudge,^5^ Jorge Ruiz-Orera,^6^ Michal Bassani-Sternberg,^7,8,9^ Robert L. Moritz,^10^ Eric W. Deutsch,^10^ Sebastiaan van Heesch^4^

**Affiliations**

^1^Department of Pediatrics, Division of Pediatric Hematology/Oncology, University of Michigan Medical School, Ann Arbor, MI 48109, USA

^2^Department of Biological Chemistry, University of Michigan Medical School, Ann Arbor, MI 48109, USA

### ^3^Broad Institute of MIT and Harvard, Cambridge, MA, 02142, USA

^4^Princess Máxima Center for Pediatric Oncology, Heidelberglaan 25, 3584 CS, Utrecht, the Netherlands

^5^European Molecular Biology Laboratory, European Bioinformatics Institute, Wellcome Genome Campus, Hinxton, Cambridge CB10 1SD, UK

^6^Cardiovascular and Metabolic Sciences, Max Delbrück Center for Molecular Medicine in the Helmholtz Association (MDC), 13125 Berlin, Germany

^7^Ludwig Institute for Cancer Research, University of Lausanne, Agora Center Bugnon 25A, 1005 Lausanne, Switzerland

^8^Department of Oncology, Centre hospitalier universitaire vaudois (CHUV), Rue du Bugnon 46, 1005 Lausanne, Switzerland

^9^Agora Cancer Research Centre, 1011 Lausanne, Switzerland

^10^Institute for Systems Biology (ISB), Seattle, Washington 98109, USA

**Address correspondence to:**

John R. Prensner, MD, PhD

Department of Pediatrics and Biological Chemistry

Medical Science Research Building II, Room 2560B

1150 Medical Center Drive

Ann Arbor, MI 48109

Email: [prensner@umich.edu](mailto:prensner@umich.edu)

Phone: 734-763-5939

**List of Supplementary Tables**

Supplementary Table 1: Description of aggregated data

Supplementary Table 2: A description of ORF primary datasets and data sources

Supplementary Table 3: Phase I Ribo-seq ORFs detected in only 1 of 8 studies

Supplementary Table 4: A metaanalysis of non-canonical ORFs present in 8 different high-stringency datasets

Supplementary Table 5: ORFs included for this analysis from the Duffy et al. dataset.

Supplementary Table 6: ORFs included for this analysis from the Ouspenskaia et al. dataset.

Supplementary Table 7: The number of ORFs for comparative analysis across studies

Supplementary Table 8: The number of ORFs per sample and per cell/tissue type according to each study
